# Modulation of expression and cellular distribution of p21 by macrophage migration inhibitory factor

**DOI:** 10.1186/1476-9255-6-24

**Published:** 2009-08-24

**Authors:** Elliott Taranto, Jin R Xue, Eric F Morand, Michelle Leech

**Affiliations:** 1 Centre for Inflammatory Diseases, Monash University Department of Medicine, Monash Medical Centre, Clayton, Melbourne, Australia

## Abstract

**Background:**

The pleiotropic protein MIF, (macrophage migration inhibitory factor), has been demonstrated to modulate several key proteins governing cell cycle control and is considered to contribute to cell growth and differentiation. In this study we investigated the effect of MIF on the expression and cellular distribution of the CDK inhibitor p21.

**Methods:**

The effect of endogenous MIF on p21 expression and distribution was examined by comparing murine dermal fibroblasts derived from *wt *and MIF -/- mice. The effect of MIF on cell growth and apoptotic rates was compared using ^3^H-Thymidine incorporation assays and annexin V/PI assays respectively. Total p21 protein levels were compared using flow cytometry and western blotting. p21 mRNA was assessed by RT-PCR. Intracellular p21 staining was performed to assess cellular distribution of total protein. To further confirm observations siRNA was used to knockdown MIF protein in *wt *cells. Cell cycle analysis was performed using PI incorporation assays.

**Results:**

MIF-/- murine dermal fibroblasts exhibited reduced proliferative responses and were more susceptible to apoptosis. This was associated with reduced p21 expression and nuclear distribution. Treatment with recombinant MIF protein was demonstrated to reduce both basal and induced apoptosis and increase nuclear p21 expression. Reduced nuclear p21 expression was also observed in MIF siRNA treated *wt *cells.

**Conclusion:**

The results demonstrate that in the absence of MIF p21 expression and nuclear distribution is reduced which is associated with a reduction in cell growth and increased apoptosis. MIF may therefore play a role in maintaining homeostatic control of p21.

## Background

Phase transition of the cell cycle is dependent upon the ordered activation and subsequent assembly of cyclin-dependent kinases (CDKs) with their regulatory subunit proteins, cyclins. Quiescent cells are stimulated to produce cyclins in response to specific mitogenic signals. The pro-proliferative activity of cyclin-CDK complexes are tightly regulated by CDK- inhibitors (CKIs) that block the catalytic activity of the holoenzymes. P21 is a member of the Cip/Kip family of CKIs which also consists of p27 and p57. p21 is therefore traditionally identified as a cell cycle arrest protein. Its function in the p53 tumour suppressor pathway as an inhibitor of G1 CDK's resulting in cell cycle arrest in response to various cellular stresses is well described in the literature [1,2]. However, more recent investigations have revealed new roles for p21 in normal cell cycle progression [3-6] and in active proliferation [7,8].

Recent studies have shown that the proinflammatory cytokine macrophage migration inhibitory factor (MIF) is an important contributor to normal cell division as well as oncogene-induced malignant transformation and tumorigenesis [9]. One mechanism through which MIF may exert these effects is via escape from p53 mediated cell cycle control. This concept is supported by studies from our lab wherein recombinant MIF (rMIF) treatment leads to decreased p53 protein expression as measured by Western blotting [10]. MIF mediated escape from p53 effect is also demonstrated in studies by Hudson et al where the addition of either purified recombinant MIF or over-expression of an ectopically expressed MIF could overcome p53-induced growth arrest, senescence and apoptosis [11]. Similarly, MIF has shown to sustain the survival of macrophages through suppression of p53 [12].

In this study we find that the absence of MIF impacts significantly on the expression and cellular distribution of p21. This observation was associated with a marked reduction in cell growth and increased apoptosis, suggesting a role for MIF in maintaining normal homeostatic control of p21 expression and distribution.

## Methods

### Animals

MIF-/- mice were generated via homologous recombination in J1 embryonic stem cells as described previously [13]. They were maintained on a mixed background of 129/Sv C57BL/6. MIF+/+ wild-type (*wt*) mice of the same background were bred from MIF-/- littermates and used as controls. Mice were housed in a conventional housing facility. All animal experiments were performed in accordance with the regulations of Monash University Animal Ethics Committee.

### Isolation and culture of murine dermal fibroblasts

Murine dermal fibroblasts (MDF) were obtained from MIF-/- and *wt *mice. Dermal fibroblasts were isolated by dissection of the dermal layer. A single cell suspension was obtained by digesting minced dermal tissue with 2.4 mg/ml Dispase (grade II, 5 U/mg; Boehringer Mannheim, Melbourne, Australia), 1 mg/ml collagenase (type II; Sigma, Melbourne, Australia) and DNase (type I; Boehringer Mannheim). MDF were propagated at a cell density of 1-5 × 10^6 ^cells per 10 cm culture plates in RPMI (ICN Biomedicals, Cincinatti OH)/10% FCS (ICN Pty Ltd, Melbourne, Australia) at 37°C in a 5% CO_2 _humidified incubator. Cells were used between passages 4 and 14. Comparisons between MIF-/- and *wt *cells were made using cells of the same passage.

### Fibroblast proliferation by ^3^H-Thymidine assay

MDF were seeded onto a 24 well plate at a density of 5 × 10^4 ^cells/well (70% confluence) in RMPI (10%FCS) and incubated for 24 hours at 37°C. Cells were then washed twice with Hanks buffered saline solution (HBSS) before starving in 500 ul RPMI (0.1%BSA) at 37C for 24 hours and were then cultured for 30 hours in 10% FCS. Cells were pulsed with 1 uCi/ml ^3^H/Thymidine in 50 ul RPMI for 18 hours at 37°C. Radioactivity content was determined by scintillation counting using a Wallac 1409 Liquid Scintillation Counter (3H/Thy, 60 seconds/tube). DNA synthesis was estimated by measurement of ^3^H-Thy incorporation in fibroblasts. Results were expressed as the average CPM count with the standard error of the mean.

### Fibroblast proliferation by cell count

*Wt *and MIF-/- MDF were seeded onto a 24 well plate at a density of 5 × 10^4 ^cells/well (70% confluence) in RMPI (10%FCS) and incubated for 72 hours at 37°C. At 24 hour time points, cells were washed with Hanks buffered saline solution (HBSS) and removed by trypsinisation. A single cell suspension was prepared 1:1 ratio with trypan blue (Sigma) and counted using a haemocytometer. Results were expressed as the total number of viable cells (cell count).

### Fibroblast apoptosis

Murine Dermal Fibroblasts (MDF) seeded onto a 6 well plate at 2 × 10^5 ^cells per well in RPMI (10% FCS) and allowed to adhere overnight. After 24 hours non adherent cells were washed off in HBSS and each well was replenished with RPMI (10% FCS) with or without SNP (0.50 mM)/(0.25 mM) and rMIF (100 ng/ml) treatment. 24 hours later apoptotic cells were differentiated by combined application of annexin V-FITC and PI. Briefly, cells were trypsinised, centrifuged and re-suspended in binding buffer (10 mM HEPES/NaOH, 0.14 M NaCl, 2.5 mM CaCl2, pH 7.5). Samples were incubated with annexin V-FITC and PI for 30 min at room temperature and analysed by flow cytometry. Annexin V-FITC and PI fluorescence was detected in the FL-1 (green) and FL-2 (red) channels respectively after correction to the spectral overlap between the two channels. Apoptotic and necrotic cells were distinguished on the basis of annexin V-FITC reactivity and PI exclusion. Live, non-apoptotic cells were not stained with any of the reagents. Apoptotic cells exhibited intense green (FITC) and low or intermediate red (PI) fluorescence (early and late stages of apoptosis, respectively). Permeability of late apoptotic cells for PI is the consequence of the compromised integrity of their plasma membrane. Results are expressed as % apoptosis, the percentage of annexin V/PI and annexin V positive cells relative to total number of cells gated.

### Detection of P21 in FLS by Western blotting

P21 expression in *wt *and MIF-/- MDF were compared using Western blotting with a monoclonal antibody specific for p21 (F-5 Santa Cruz, CA). Briefly, cells taken from semi confluent (70%) culture were washed with cold PBS and then lysed with 2× SDS sample buffer. The protein samples were boiled for 10 minutes and stored at -20°C. Samples were subjected to 10% Tris Glycine iGel SDS/PAGE (Gradipore, Sydney Australia), transferred to a Hybond C membrane and detected using ECL detection system (Amersham).

### RNA extraction and real-time polymerase chain reaction (RT-PCR) analysis

Total RNA was extracted from MDF using TRIzol reagent (Gibco BRL, Grand Island, NY), according to the manufacturer's protocol. Two micrograms of total RNA was reverse transcribed using Superscript II reverse transcriptase and oligo (dT)18 (Gibco BRL). PCR amplification was performed on a LightCycler (Roche Diagnostics, Castle Hill, Australia) by using SYBR Green I as a double-stranded DNA-specific binding dye and continuous fluorescence monitoring. MIF, p21 and β-actin PCR products were purified on agarose gel electrophoresis using the QIAEX II gel extraction system (Qiagen, Clifton Hill, Australia). The level of expression of messenger RNA (mRNA) of MIF and or p21 was determined relative to the standard preparation using the LightCycler computer software.

For PCR, 5 μl each of the standard and the sample complementary DNA dilutions were added to individual capillary tubes. Amplification was carried out in a total volume of 10 μl containing primer concentrations of 3 pmoles and 1 μl of dNTP mix, Taq, reaction buffer, and SYBR Green I dye as supplied in the LightCycler DNA Master SYBR Green I kit (Roche). Forty cycles of PCR were programmed. Relative quantification of target mRNA expression was calculated and normalized to the expression of β-actin. The results, based on the ratio of target mRNA to β-actin amplification, are presented as the fold induction in mRNA expression relative to the amount present in control samples.

### Detection of p21 in murine fibroblasts by immunofluorescence

MDF were seeded on mini-slides placed within a 24 well plate at a semi confluent density (70%) of 2.5 × 10^4 ^cells per slide for 24 hours. Adherent cells were then washed with HBSS, and fixed in 2% paraformaldehyde. Cells were again washed and then permeabilised in 0.1% Triton X (Sigma), or 0.1% Saponin (Sigma) for extranuclear staining, before being washed again in PBS/0.1% Na azide/0.1% BSA and incubated with specific monoclonal antibody to p21 (F-5) or isotype-matched negative control antibodies (Santa Cruz, CA) for 30 minutes. Unbound antibody was removed by washing in PBS/0.1% Na azide/0.1% BSA. Cells were then incubated for 30 minutes with FITC conjugated-secondary antibody (Silenus, Melbourne, Australia) before being washed and analysed under UV microscope.

### Detection of p21 in murine fibroblasts by flow cytometry

MDF taken from semi confluent culture were washed with HBSS, and fixed in 2% paraformaldehyde. Cells were again washed and then permeabilised in 0.1% Triton X (Sigma) for 30 minutes before being washed again in PBS/0.1% Na azide/0.1% BSA and incubated with specific monoclonal antibody to p21 (F-5) or isotype-matched negative control antibodies (Santa Cruz, CA) for 30 minutes. Unbound antibody was removed by washing in PBS/0.1% Na azide/0.1% BSA. Cells were then incubated for 30 minutes with FITC conjugated-secondary antibody (Silenus, Melbourne, Australia) before being washed and analysed by flow cytometry. Results are expressed as delta mean fluorescence intensity (ΔMFI)

For intracellular analysis of p21 protein by flow cytometry 0.05% Saponin was used to permeabilise the cell wall leaving intracellular membranes intact. As above cells were incubated with p21 antibody or negative control antibody and analysed by flow cytometry. Nuclear p21 was estimated by subtracting ΔMFI of cytoplasmic p21 from ΔMFI of total p21.

### SiRNA

RNA interference was used to knockdown MIF protein expression by introducing a homologous double-stranded RNA. The nucleotide sequences of dsRNA and complimentary dsRNA for mouse MIF mRNA were 5'-CCGCAACUACAGUAAGCUGdTdT-3' and 5'-CAGCUUACUGUAGUUGCGGdTdT-3', respectively. As a control RNA duplex 5'-GCGCGCUUUGUAGGAUUCGdTdT-3' and 5'-CGAAUCCUACAAAGCGCGCdTdT-3' were used. MDF grown in RPMI 1640 with FCS (10%) were transfected with either the MIF siRNA or the control RNA duplex using Amaxa nucleofection electroporation (Amaxa AG, Germany) according to the manufacturer's protocol [14]. After 24 hours, cells were transfected with either MIF siRNA or the control siRNA a second time. 24 hours after the second transfection the culture medium was removed and cells were examined for p21 immunofluorescence.

### Cell cycle analysis

MDF taken from semi confluent culture via HBBS wash/trypsinisation were resuspended in PBS before being resuspended in ethanol and left on ice for 2 hours. The suspension was then centrifuged and ethanol thoroughly decanted. The pellet was resuspended in a staining solution of 0.1% triton-X, DNase free RNase and Propidium iodide (PI) for 30 minutes at room temperature. Samples were then analysed by flow cytometry.

### Data Analysis

ΔMFI was calculated by subtracting the mean fluorescence of the samples stained with an isotype matched negative control antibody from that of the samples stained with specific antibodies. Results are expressed as the mean +SEM. Statistical analysis was performed using the Students t test. P < 0.05 was considered statistically significant.

## Results

### MIF-/- cells exhibit reduced proliferative responses

**Figure 1:**
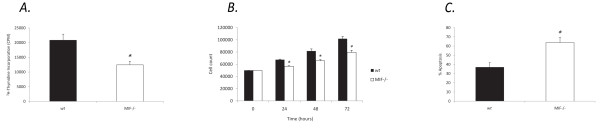
**MIF-/- cells exhibit reduced proliferative responses and increased apoptosis**. **a**. Quantification of basal MIF-/- and *wt *MDF proliferation by ^3^H-thymidine incorporation. Significantly lower levels of thymidine incorporation were observed in MIF-/- MDF compared *wt *MDF, (N = 5, * P < 0.05). **b**. Proliferation of MIF-/- and *wt *MDF further quantified by haemocytometric cell count over 72 hours (N = 4, * P < 0.05). **c**. Apoptosis of MDF in response to cellular stress induced by 0.5 mM SNP was assessed by flow cytometric analysis of annexin V-FITC and PI. Percentage apoptosis represents all non-viable cells, (N = 4, * P < 0.05).

To determine the role of endogenous MIF in cell growth we examined proliferation in MIF-/- and *wt *MDF using a ^3^H-thymidine incorporation assay and cell count assay. After a 42 hour analysis of 6 independent experiments we found that cells lacking endogenous MIF displayed significantly reduced cell growth, (12437.9 +/- 1253.3), compared to *wt *MDF (20841.7 +/- 2040.6) (Figure 1a). This was confirmed by cell count assays which revealed significantly reduced numbers of viable MIF-/- cells compared to *wt *over 24, 48 and 72 hours (Figure 1b).

### MIF-/- cells are more susceptible to apoptosis

To further characterise the effects of endogenous MIF on cell cycle profile, we examined apoptotic events in MIF-/- and *wt *MDF by annexin V-FITC and PI dual labeling. Compared with *wt *MDF, MIF-/- MDF were more prone to undergo cell death exhibiting increased SNP induced apoptosis, (63.8 +/- 5.5), compared to *wt *MDF, (36.7 +/- 5.2) (Figure 1c).

### rMIF rescues cells from basal and SNP induced apoptosis

**Figure 2:**
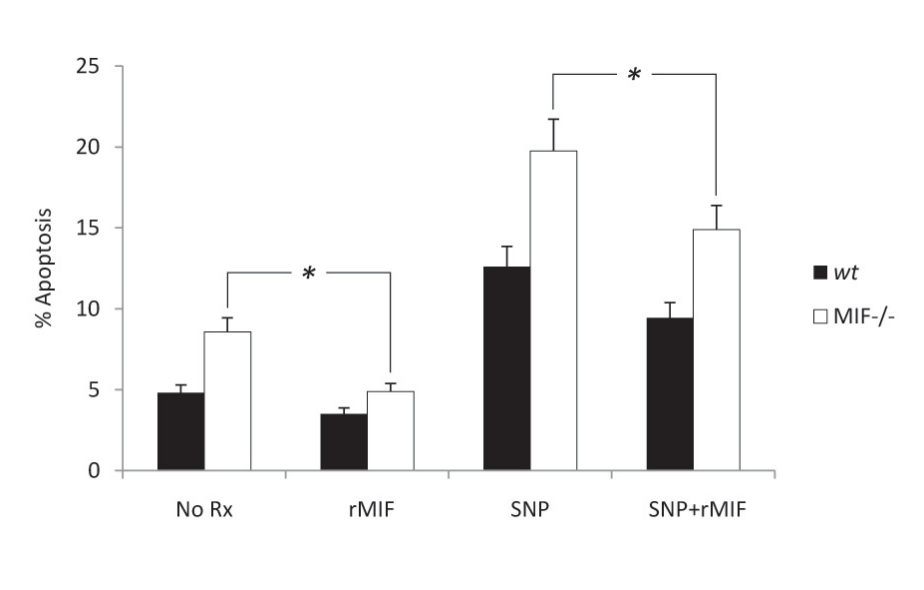
**rMIF rescues cells from basal and SNP induced apoptosis**. The effect of rMIF on apoptosis of MDF was examined using cytometric detection of annexin V/PI dual labeling. MIF-/- MDF demonstrated increased rates of spontaneous apoptosis which was reduced following treatment with 100 ng/ml rMIF. Similarly, rMIF treatment abrogated SNP (0.25 mM) induced apoptosis in both MIF-/- and *wt *MDF, (N = 3, * P < 0.05).

To explore the potential for exogenous MIF to alter cell cycle events we examined both basal and induced apoptotic events in MIF-/- and *wt *MDF with and without rMIF treatment. Compared with control, treatment with rMIF protein was demonstrated to significantly increase basal survival and to significantly reduce SNP-induced apoptosis in MIF-/- MDF (Figure 2).

### p21 expression is reduced in MIF-/- MDF

**Figure 3:**
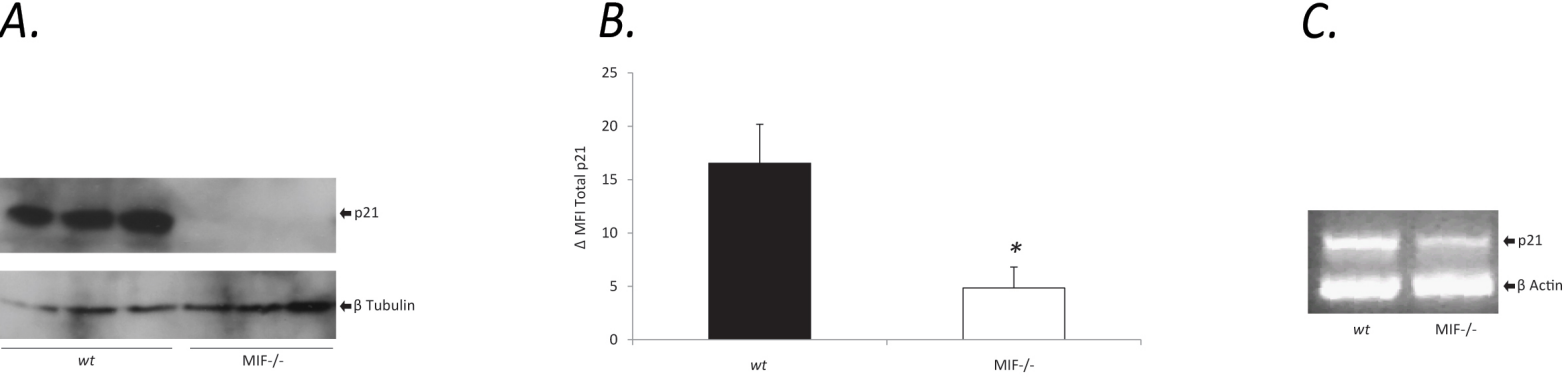
**Reduced p21 expression in MIF-/- cells compared with *wt***. **a**. Levels of p21 expression in MIF-/- and *wt *MDF assessed by Western blotting using a monoclonal antibody to p21. The equivalence of protein loading was measured by detection of β-tubulin. Each lane represents a single MIF-/- or *wt *MDF mouse. **b**. Flow cytometric detection of p21 protein in untreated MDF taken from semi-confluent culture. Results are representative of 4 independent experiments, each measuring at least 5000 events. Expressed as delta mean fluorescence intensity (ΔMFI) which is the difference between the MFI of tested cells and the MFI of background staining (N = 4, * P < 0.05). **c**. Quantification of p21 mRNA to β-actin in MIF-/- and *wt *MDF by reverse transcription polymerase chain reaction (RT-PCR).

To examine the role of endogenous MIF in the regulation of p21 levels, MIF-/- and *wt *MDF expression of p21 was analysed using Western blotting and flow cytometry analysis. MIF-/- cells expressed lower levels of p21 protein compared to *wt *MDF (Figure 3a, 3b). Accordingly P21 mRNA was reduced in MIF-/- MDF as determined by RT-PCR (Figure 3c).

### Subcellular localisation of p21 in MIF-/- MDF

**Figure 4:**
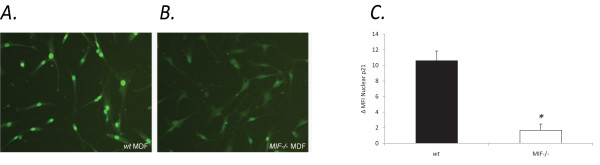
**Decreased nuclear p21 in MIF-/- MDF compared with *wt *MDF**. Immunofluorescent staining of p21 showing prominent nuclear localisation of p21 in *wt *MDF (a), in contrast to p21 expression in MIF-/- MDF showing diffuse staining in both the cytoplasm and nucleus (b). Reduced nuclear p21 protein expression was confirmed by flow cytometry (*P < 0.05) (c). In each group results are representative of 4 independent experiments.

**Figure 5:**
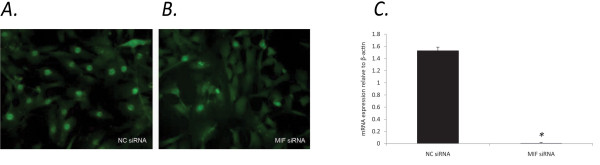
**Cells spiked with MIF siRNA display reduced nuclear p21**. In four separate experiments *wt *MDF nucleofected with MIF siRNA exhibited a significant decrease in nuclear p21 expression (b), while MDF nucleofected with control siRNA remained largely unchanged (a). The efficiency of MIF knockdown in *wt *MDF was quantified using RT-PCR. Compared with control siRNA, nucleofection of MIF siRNA leads to undetectable MIF mRNA in MDF as determined by RT-PCR (c) (N = 4, * P < 0.05).

To further explore the potential for MIF to influence p21, we examined the subcellular localisation of p21 in MIF-/- and *wt *MDF. Interestingly, MIF-/- MDF exhibited diffuse, predominantly cytoplasmic p21 staining (Figure 4b) compared to *wt *MDF, which displayed strong nuclear staining for p21 (Figure 4a). Flow cytometric analysis of nuclear p21 protein revealed a significant reduction in MIF-/- compared to *wt *MDF (Figure 4c). Similar results were seen in *wt *MDF spiked with MIF siRNA which exhibited a significant reduction in nuclear p21 (figure 5b) compared to control siRNA (Figure 5a). The efficiency of MIF knockdown in *wt *MDF was quantified using RT-PCR. After 40 cycles of amplification MIF mRNA was not detectable in samples transfected with MIF siRNA (figure 5c).

### rMIF induces p21 expression & nuclear localisation in MIF-/- MDF

**Figure 6:**
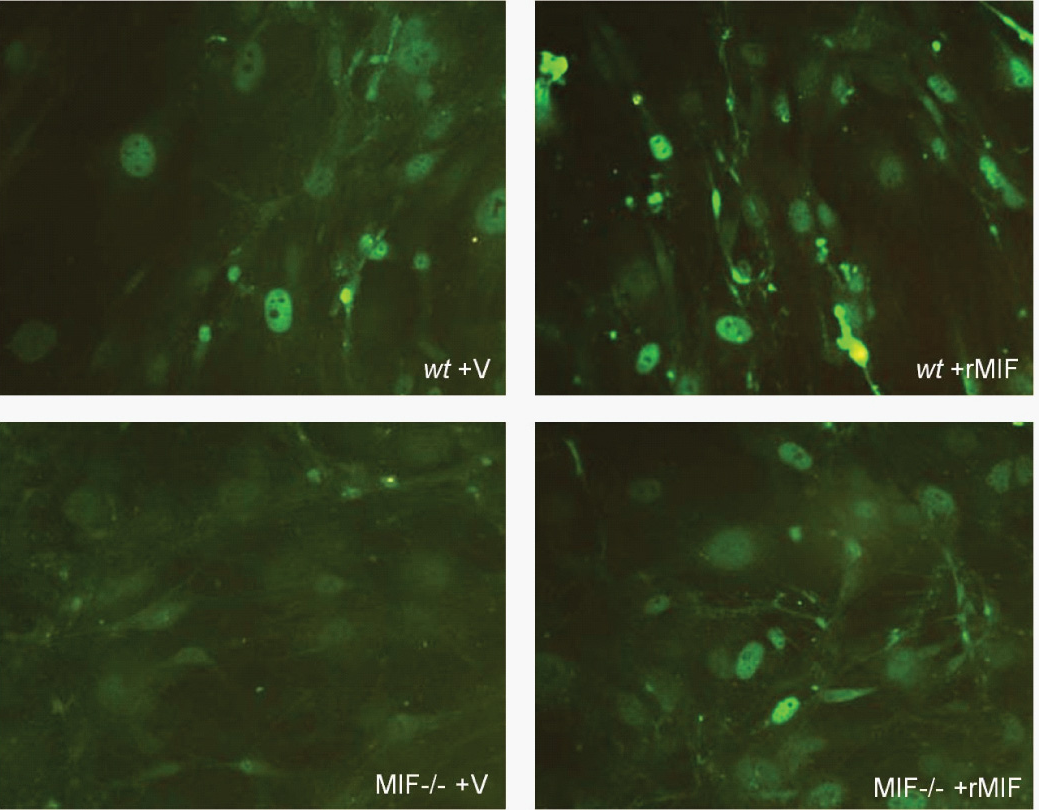
**rMIF treatment of MIF-/- MDF induces p21 expression and nuclear localisation**. No difference in p21 immunofluorescence was observed when MIF replete *wt *MDF were treated with rMIF. In contrast, compared with vehicle (V), rMIF treated MIF-/- MDF exhibited increased expression and nuclear localisation of p21 (N = 4).

To further confirm the involvement of MIF in p21 redistribution, MIF-/- and *wt *MDF were treated with rMIF and examined for p21 by immunofluorescence. Randomly cycling *wt *MDF from semi-confluent culture exhibited predominantly nuclear localisation of p21 and rMIF treatment of these cells did not yield any further increase in nuclear p21 expression. However compared to vehicle, rMIF induced a shift from predominantly cytoplasmic to nuclear localisation of p21 protein in MIF-/- MDF demonstrating MIF involvement in induction and nuclear translocation of p21 protein (Figure 6).

### P21 redistribution is not the result of cell cycle phase differences

**Figure 7:**
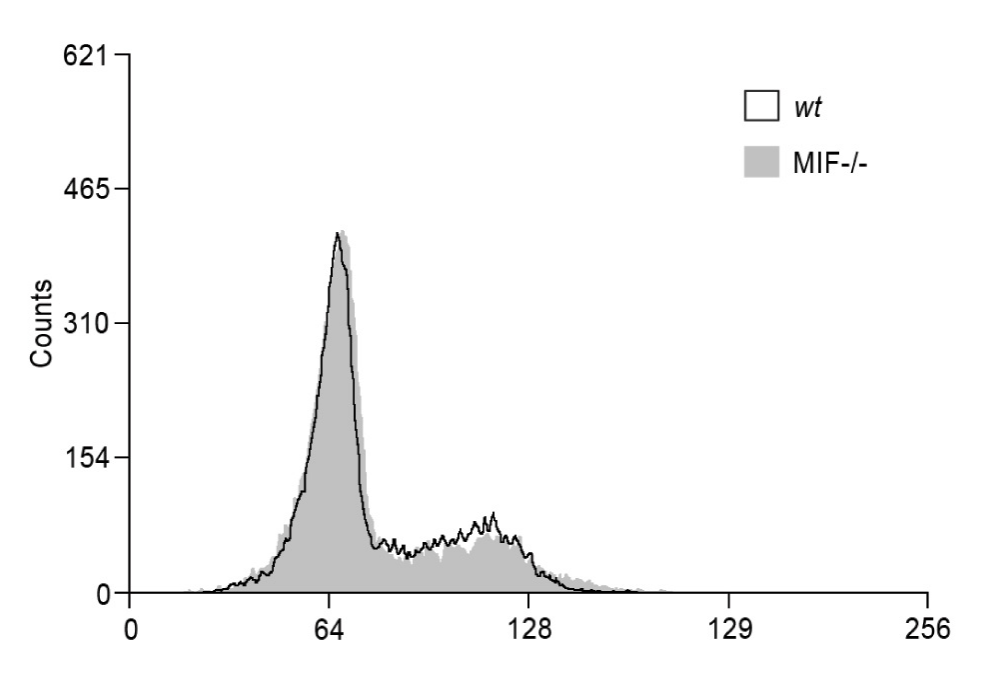
**p21 localisation is not caused by cycle phase differences in MIF-/- compared with *wt***. Cell cycle phase assessment using flow cytometric assessment of PI incorporation. Compared with *wt*, MIF-/- MDF exhibit no significant difference in the percentage of cells in each cell cycle phase. Histogram is representative of 4 experiments.

Cell cycle phase is well described to alter expression of key cell cycle proteins including p21. To address this issue, we examined the cell cycle phase profile of MIF-/- and *wt *MDF taken from semi-confluent culture using flow cytometric analysis of PI incorporation. No significant difference in the percentage of cells in each phase of the cell cycle was seen. Therefore the difference in p21 expression and subcellular localisation observed between MIF-/- and *wt *MDF could not be explained by cell cycle phase differences between the cells in these experiments (Figure 7).

## Discussion

MIF is a pro-inflammatory cytokine which is expressed constitutively by almost all cell types and is significantly upregulated in the setting of inflammatory stimulation [15,16]. The findings in this study suggest that MIF is involved in the promotion of normal cell cycle and proliferative responses in murine dermal fibroblasts. Supporting this, recent studies have demonstrated a functional role for MIF in normal cell division as well as cell survival and oncogene-induced malignant transformation making this cytokine an important new focus in tumour biology research [17-19]. Evidence suggests that MIF may also indirectly support tumour growth via promotion of angiogenic responses [20,21]. Other studies have shown that rMIF treatment or forced over expression of MIF can stimulate cell growth [22]. A number of cell types have also been shown to secrete MIF in response to growth factor stimulation [23,24]. We have previously shown dose-dependent regulation of human fibroblast proliferation by rMIF [25].

In the current study, reduced proliferative responses in MIF-/- MDF were associated with increased baseline and SNP induced apoptosis. Moreover, treatment with recombinant MIF was associated with a reduction in SNP induced apoptosis. This promotion of cell survival may have relevance to inflammatory disease in terms of persistence of inflammatory cells or growth of intrinsic cells in an inflammatory lesion. For example endogenous MIF has been shown to be important in promoting macrophage viability in the setting of inflammatory stimuli and rMIF treatment or its overexpression in a variety of cell types including fibroblasts, macrophages and endothelial cells rendered them resistant to apoptosis induced by a variety of stimuli [20,26]. Previous studies in our own laboratory have shown that rMIF treatment of human fibroblasts leads to rescue from SNP-induced apoptosis [10]. Consistent with this data, others have reported impaired DNA damage response in cells lacking MIF [27].

Our finding in this study that genetic or siRNA mediated deficiency of MIF is associated with reduced expression and/or nuclear localisation of p21 and that rMIF treatment induces nuclear localisation of p21 must be interpreted in the setting of emerging new literature regarding this protein. p21 is traditionally conceptualised as a cell cycle arrest protein. p53-induced nuclear accumulation of high level p21 expression, resulting in G1 cell cycle arrest, in response to various stressful stimuli, is well described in the literature [1,2,28]. Evidence for an unexpected requirement for basal expression of p21 in normal cell cycle progression is however found in several recent studies [3-6]. p21-/- cells exhibit a failure of normal cell cycle progression which has been related to a role for p21 as an assembly factor which promotes cyclin-D1-CDK4 binding thus contributing to cellular proliferation [29]. Moreover, LaBaer et al 1997 demonstrated that p21 provides the localisation signal for cyclin-D1-CDK4 nuclear import [29]. Other studies have shown that p21 promotes nuclear accumulation of cyclin-D1-CDK4 via its ability to inhibit cyclin D1 nuclear export [4]. These studies suggest some duality in p21 function with evidence for an unexpected permissive role for p21 in normal cell cycle progression.

In addition to these findings, evidence also exists for p21 as a direct inhibitor of apoptosis, separate from its function in arrest. Studies examining targeted overexpression of P21 have shown a significant reduction in induced apoptosis by interaction with proapoptotic molecules including procaspase-3, caspase-8 and the kinase apoptosis signal-regulating kinase 1 (ASK1) [30-34]. In p21 deficient cells a significant decrease in death receptor mediated apoptosis, including FAS-dependent apoptosis, was reported [35]. Furthermore animal studies examining p21 deficient mice have demonstrated an increased susceptibility to and aggressiveness of various tumors [36-39].

The mechanism by which MIF influences the expression and nuclear localisation of the cell cycle protein p21 requires further elucidation. Several possibilities are supported in the current literature. The potential for MIF to impact upon cell cycle via its well described down-regulation of the cell-cycle protein p53 is the subject of many previous studies [10,11,40,41]. If however the impact of MIF on p21 is entrained by p53, MIF deficiency with its attendant increase in p53 protein expression would be expected to result in increased basal p21 expression. Accordingly rMIF treatment of cells leading to decreased p53 expression would be expected to lead to decreased p21 expression and nuclear localisation. Our results are in contrast to this and suggest a p53-independent effect of MIF on p21. Clearly p53 has a powerful impact on both p21 and cell cycle but this may be more relevant in the setting of immune or oxidative stress rather than during normal proliferative responses.

We and others have shown MIF-dependant activation of ERK MAP kinase pathways and reduced ERK and other MAPK phosphorylation in cells derived from MIF-/- cells [25,42,43]. Sustained activation of MAPK is known to be important in cell cycle progression in response to particular stimuli especially growth factors. For example ERK pathway activation is required for p21 induction in vascular smooth muscle cells in response to PDGF [44] and in keratinocytes in response to TGFb [45]. Further evidence of MAPK dependant expression of p21 comes from studies using specific ERK inhibitors [46]. There is also evidence to suggest that MAPK activation leads to phosphorylation of specific sites on p21 [47].

Studies have shown that MIF is involved in adhesion dependant signaling and facilitates cell cycle progression via transcriptional regulation of cyclin D levels [48]. Additionally it has been demonstrated that MIF-mediated induction of ERK leads to cyclin D1 transcription [49]. It is therefore conceivable that p21 expression and nuclear localisation are influenced by the reduction in cyclin D, specifically the reduction in available cyclin D to complex with p21 and CDK2 which would promote nuclear retention of all three molecules.

The cytokine MIF is increasingly appreciated to play a role in oncogenic transformation and the promotion of tumor growth in addition to a broad spectrum of pro-inflammatory actions in a range of tissues and inflammatory diseases. Its validity as a target for therapeutic blockade in a spectrum of immune-mediated disease in humans is now well established [50]. Given the broad spectrum of effects MIF has on the cell cycle, it cannot be assumed that our observation of reduced proliferation and increased cell death in the absence of MIF are attributable to modulation of p21 alone. It is however feasible that it may be a contributing factor to both. The findings in this study suggest that endogenous MIF may be important in the facilitation of normal cell growth and that MIF promotes growth responses and cell viability both basally and in the setting of oxidative stress, such as SNP. This may be of relevance in the assessment and application of MIF blockade strategies. The finding of reduced p21 expression and nuclear localisation in MIF-/- mice and the induction of p21 nuclear localisation by MIF suggest a novel mechanism, namely the maintenance of basal nuclear p21 levels, by which MIF mediates its permissive effects on cell growth and cell cycle progression.

## Abbreviations

CDK: Cyclin-Dependent Kinase; ERK: Extracellular Signal-Regulated Kinase; MAPK: Mitogen-Activated Protein Kinase; MIF: Macrophage migration Inhibition Factor; PDGF: Platelet-Derived Growth Factor; TgF: Transforming Growth Factor; SNP: Sodium Nitroprusside; PI: Propidium Iodide.

## Competing interests

The authors declare that they have no competing interests.

## Authors' contributions

JRX carried out the RT PCR and western blotting studies. ET carried out all other studies and drafted the manuscript. ML conceived of the study and helped draft the manuscript. Both EFM and ML participated in the design and coordination of the study. All authors read and approved the final manuscript.

## Author information

Elliott Taranto BSc (Hons)

Jin Rong Xue, MD

Eric F. Morand MBBS (Hons) FRACP PhD

Michelle Leech MBBS (Hons) FRACP PhD
